# The Hospital Nursing Supplement

**Published:** 1894-07-14

**Authors:** 


"The Hospital" Nursing Mirror.

Being the Extra Nursing Supplement of "The Hospital" Newspaper.

[Contributions for this Supplement should be addressed to the Editor, The Hospital, 428, Strand, London, W.C., and should have the word "Nursing" plainly written in left-hand top corner of the envelope.]

### News from the Nursing World.

### THE ROYAL CHRISTENING.

Monday next, July 16th, has been named for the christening of the infant son of their Royal Highnesses the Duke and Duchess of York. The Queen intends to be present at the service, which will take place at White Lodge, and will be performed by the Archbishop of Canterbury.

### PRINCESS BEATRICE AND THE QUEEN'S NURSES.

H.R.H. Princess Henry of Battenburg has promised to visit Southampton at the end of July for the purpose of presiding at a meeting in connection with the local branch of the Queen's Jublilee Institute for Nurses. Much valuable work has already been done amongst the sick poor in their own homes, and funds are urgently needed for extending the field of the nurses' labours and also for increasing the number of the district nurses. Her Royal Highness has consented to receive purses containing three guineas and upwards on behalf of the Home.

### VIOLENCE TOWARDS A PAUPER.

The Holborn Board of Guardians have dismissed "Nurse" Greengo for striking an aged pauper who was under her charge in the workhouse. There was apparently no doubt as to the bruises on the old woman's face having been caused by the blow, yet two of the Guardians, as reported in the Press, disagreed with the recommendation of the Mitcham Workhouse Committee and favoured the idea of retaining the nurse. One gentleman thought dismissal "a very severe punishment" and spoke of "extenuating circumstances," whilst another is reported as saying "that the sentence of the committee was more than was warranted by the offence." Miss Baker courageously championed the cause of the poor, asserting that there were no "extenuating circumstances" and that it was not the first time she had heard "a case of this kind" defended there. It is to be hoped that if not the first it will, at any rate, be the last time that "guardians of the poor" will be found attempting to justify harsh treatment of the paupers for whom they are responsible.

### THE ATTRACTIONS OF LAWN TENNIS.

Tennis balls and bats have been purchased for the nurses at Sheffield Workhouse Infirmary, and one of the guardians has stigmatised this expenditure as "abominable," according to the report of the board meeting given in the *Sheffield Daily Telegraph*. Another gentleman defended the transaction, which, he said, had been considered by the hospital committee, on the ground of economy. He believes that the attractions of the popular game will go far to make the nurses "more contented with their lot," and thus the guardians will be spared the expense of advertising for new nurses. This is indeed a novel aspect of the transaction, and one so economical as ought to commend it to the guardians. If the opposition raised was solely on account of "the ratepayers' pockets" the objector has a remedy in his own hands. The gift of a complete tennis set presented by himself as a private personal offering would be a graceful act, to which exception could be taken by no one.

### OUR SOLDIERS' WIVES.

Two district nurses are stationed at Aldershot, through the kindly agency of the Soldiers and Sailors' Families' Association. A special fund supports them, and they "can be requisitioned for attendance on families needing them, both in and off the strength." It is also satisfactory to learn that a notice and recommendation of their presence are contained in the divisional orders.

### SICK PAUPERS AT BATH.

At the meeting of the Bath Guardians last week a discussion took place as to the action of the master in refusing admittance to a doctor who had obtained permission from a member of the board to go over the infirmary. As reported in the local press, the master chiefly defended his refusal on the grounds of suspecting the visitor of being connected with a medical journal. The valuable reports on other infirmaries already published by the *British Medical Journal* show that other workhouses are less afraid of inspection, and it is to be hoped that a day will soon come when every infirmary in England will be open as a matter of course to interested visitors at reasonable hours. Whilst it lies in the power of the master to keep the sick poor as isolated as if they were criminals there is little hope of their lots being substantially improved. Dr. Craddock's constant advocacy of the claims of paupers to proper nursing is bearing fruit at Bath in the appointment of a night nurse. Hitherto pauper inmates have been the only attendants on the sick during the night, and it is said that the female attendant gets precarious repose by going to bed in the day in the ward in which her duty lies. But even this amount of rest is not granted to the male inmates, who are supposed, as reported in the *British Medical Journal*, to watch the sick men by night, and be about in the day. The same report mentions the absence of any supply of hot water in the infirmary, and therefore it is hardly likely that the incident was unique which Dr. Craddock reported, of a linseed poultice being left for twenty hours on a child with pneumonia. The only hot water obtainable during the night for all purposes being contained in a kettle, work must have been inadequately performed even had trained nurses been present. Our readers will cordially agree with the sentiment that, "until we can get medical men and trained nurses as inspectors of workhouse infirmaries, publicity is the pauper's one protection and the press is the poor man's real guardian."

### HIDDEN PERILS.

The severe tests which the quality of ice creams sold in the streets are now undergoing through the agency of the British Institute of Preventive Medicine seems in no way to interfere with the present enjoyment of these delicacies by the juvenile population. If ices here should be proved to contain anything like the offensive ingredients of those recently examined in Paris, which, in the words of the *British Medical Journal*, "simply swarm with microbes of all kinds, besides containing fæcal matters and other disgusting impurities," steps should immediately be taken to protect the children who chiefly patronise them. Rotten fruit, equally in favour on account of cheapness, is another of the hidden perils which haunt the path of unwary infants in the London streets, and it is probably an even more difficult one to guard against. The results of such deleterious dieting come within the special experience of district nurses.

### ROYAL INFIRMARY, DERBY.

The excellent condition of the wards and other portions of the new buildings opened by the Duke and Duchess of Devonshire on Saturday last eloquently testified to the efficiency and zeal of Miss Carvosso, the matron, and her staff, who must have laboured hard indeed to have brought everything into order. On Saturday morning Miss Carvosso was presented by the nurses with a silver purse of beautiful workmanship, a fact which proves the kindly spirit which characterises the life within the infirmary itself. We hope that the committee may soon see their way to affiliate the infirmary to the Royal National Pension Fund for Nurses, and so secure an adequate provision for the whole of their staff at about one-third of the actual cost of the benefits which will be thus obtained.

### TIMELY ADVICE.

The Holywell Board of Guardians have agreed to engage an extra trained nurse for their infirmary, in accordance with the recommendation of Mr. Bircham, the Government Poor Law Inspector. His other admirable suggestions have been referred to the Visiting Committee, by whom they will doubtless receive the consideration they merit. Amongst other things needing reform seem the medicines, which are left lying about, in consequence of there being no proper cupboard for them, which is particularly undesirable in the case of imbeciles. The day rooms referred to as repulsive to a decent man evidently need to be greatly improved, and Mr. Bircham spoke forcibly to the Board of Guardians on the need of humanising influences and good nursing being provided for the aged and sick poor.

### COMPULSORY PRAYERS.

A complaint was made in Paris the other day, at a meeting of the Municipal Council, to the effect that a little child in the Salpêtrière Hospital had been required by the nurse and the priest to repeat prayers to which her parents, being Freethinkers, objected. A similar complaint was brought forward with regard to a nurse at the Tenon Hospital. At all the Parisian hospitals "freedom of conscience" is expressly permitted, and although all attempts at proselytising are discouraged, every facility is said to be given to patients who wish to be visited by a priest.

### QUEEN'S NURSE AT DROGHEDA.

The Boyne Weaving Company, which owns the Right Hon. T. A. Dickson as its head, has for the past six months maintained a Queen's Nurse for the sick poor of Drogheda. An appeal is now being made for public support, and if this receives a liberal response it is thought that the services of a second nurse may be obtained through the Queen's Jubilee Institute.

### "SISTERS OF THE PEOPLE."

The Calvinistic Methodists have been holding a conference at Cardiff under the auspices of "the Forward Movement." They find that their male missionaries are unable to cope with "certain phases of evils and suffering," and are therefore considering the possibility of forming a "Sisters of the People department." The establishment of a training home for nurses forms an important feature in the scheme, and it is doubtless under contemplation to give the "Sisters" a thoroughly practical education in every branch of trained nursing.

### OPHTHALMIA AND ECZEMA.

At a meeting of the Hackney Guardians on the 27th June Mr. Fenton Jones, the chairman, is reported to have said "that, as far as the board was concerned, they had always done their duty to the children in the schools," and he "utterly repudiated the assertion" made by Mr. Justice Day that they "had done it in a most perfunctory way." At the present time 127 cases of ophthalmia and 17 of eczema capitis are reported by the medical officer as under treatment in the Hackney Schools at Brentwood. It is to be hoped that these children are tended by nurses who understand the precautions and possess the trained skill to carry out the doctor's orders intelligently.

### NURSES FOR CEYLON.

The *Ceylon Observer* states that four additional nurses are to be summoned from France to the Civil Hospital, Colombo, where the present nurses are much overworked. We gave a description of this beautifully situated institution in The Hospital of February 11th, 1893, and drew attention to the fact of the nursing being entirely in the hands of untrained Roman Catholic Sisters, the night duties all being left to special native orderlies. It is therefore to be hoped that the fresh importation of so-called nurses from France really consists of ladies of the requisite qualifications and experience of trained nurses.

### STEINHOUSEMUIR.

An excellent address was given by Miss Harrison at the meeting held at Steinhousemuir the other day, to discuss the installation of a Queen's nurse in Larbert Parish. Miss Harrison, who had come from Edinburg on purpose, explained the methods of working adopted by the Queen's Jubilee Institute, and it was finally resolved that the necessary funds should be raised, and an association be formed to secure a Queen's nurse and affiliation with the institute.

### SHORT ITEMS.

The *Zenana* for July states that there are sixteen candidates ready to go out to India in connection with the Bible and Medical Mission, three being fully qualified women doctors.—At Kalimpong, in the Himalayas, a commodious hospital has been erected by the members of the Women's Guild, connected with the Church of Scotland.

### On General Nursing.

By Rowland Humphreys, M.R.C.S., L.R.C.P.Lond.

### XX.—PERITONITIS (*continued*).

All forms of peritonitis have certain symptoms in common. These are vomiting, fever, rapid pulse, shock, rigid and tender abdomen, distension of abdomen, constipation, and a tendency to lung complications.

The vomiting, fever, rapid pulse, and lung complications are mainly due to the septic poisoning, which is a marked feature of the peritonitis. Indeed, Treves remarks that the symptoms of a person suffering from peritonitis are those of a person suffering from poisoning, and that the same general characteristics are seen in cases of cholera and similar complaints as in peritonitis. The abdominal tenderness and rigidity are indicative of the inflammatory mischief inside. It has been remarked that the chest, so far as its lower part is concerned, is supplied by the same nerves as the abdomen, and that they are closely connected with the sympathetic plexuses of nerves which supply the organs within the abdomen; the peculiarity in the respiratory movements in cases where the abdomen has inflammatory mischief going on within it is explained by these anatomical relations, the breathing being entirely confined to the chest, the abdomen remaining motionless during respiration. This is, of course, an illustration of the way in which nature tries to effect a cure by giving complete rest to inflamed structures, an immovable abdomen being very characteristic of peritonitis.

The amount of the shock varies with the severity of the onset of the attack, and is worst in those cases where it follows perforation, and where highly septic matter has been thrown into the abdomen. It appears to vary chiefly with the dose of the poison in the peritoneum. This has been experimentally proved. A small dose was shown to set up only a localised peritonitis; a larger dose set up general inflammation; a still larger dose killed in a few hours, and no sign of inflammation was to be discovered on post-mortem examination.

The signs of inflammation vary in the same way as the shock.

The vomiting comes on early, and is often the first suspicious sign. It is not usually very distressing, being only excited by swallowing food or drink. No drug appears to have any but a temporary effect on it. It often passes off for a while, only to return again in a short time. So long as vomiting continues the patient must be considered of be suffering from the peritonitis, and although when near death all other symptoms seem to improve, yet this one persists. As to its cause, it appears to be partly due to irritation of the outside and of the inside of the stomach, and partly to blood poisoning of the vomiting centre. It certainly is often found where the stomach has not been attacked by the inflammatory processes, and on the other hand it is increased by nitro-glycerine, a drug which acts almost as a certain cure in most other forms of vomiting. The rapid pulse is characteristic of peritonitis, but the pulse is also, in typical cases, very small and wiry. In general terms a pulse which is rapid, out of proportion to the respiration and to the temperature, is either due to inflammation about the heart, or to blood poisoning, and, if combined with other symptoms of peritonitis, is highly characteristic of this complaint. It may, however, be large and soft. A small rapid pulse with a dry brown tongue is always indicative of the necessity for a stimulant. Champagne, good brandy, or other alcoholic stimulant may be given in very small quantities, and is best administered per rectum.

The distension of the abdomen is due to fermentative decomposition taking place within the intestine super-added on the paralysed condition of the intestine, and of the abdominal muscles which come on at a late stage of the complaint. It is a very serious condition, partly from the pressure it exerts on the neighbouring organs, such as the heart, and partly because it indicates the general condition. It is treated by antiseptic or aperient drugs given by the mouth, by washing out the intestine, or by enemata. Frequently the enemata are retained, owing to the paralysed condition of the bowel, but the fluid and gas may be drawn off by a catheter or other soft tube if a second enema does not return. Frequently the fluid injected is absorbed. The object of the enema is to remove not only the fermenting material but also the putrefactive poisons which have a great deal to do with the symptoms of peritonitis as they are absorbed and circulate in the blood, and absorption of the fluid will assist the excretion of these poisons by acting on the kidneys. Aperients are not suitable in all cases, but in some they are of the greatest advantage, and, in the author's opinion, they are of especial value where the symptoms of blood poisoning evidently exceed those which can be accounted for by the amount of inflammation present. He recently had a case of peritonitis in an elderly woman who appeared to have been suffering from attacks of a mild form of the complaint on and off for some time. An acute attack had developed itself when he first saw her, and he put her under the usual opium treatment, as a sequence, the vomiting became stercoraceous, and the patient got into a very grave condition, the abdomen being distended, and general peritonitis having made its appearance. He then changed the opium treatment, which was not even relieving her pain, that is, was not being absorbed from the bowel, for that of "White Mixture," that is, sulphate of magnesia and carbonate of magnesia, adding some turpentine in order to assist the intestines to contract; the bowels acted freely and she at once began to improve. On several subsequent occasions he has adopted the same treatment for her, with an occasional hypodermic injection to relieve the pain, and always with the best results.

The preliminary shock has to be combated with the aid of stimulants and morphia, but no food should be given by the mouth. If thus given it remains in the stomach or intestine, little or no absorption going on from these organs in peritonitis. If it is, for one reason or another, considered desirable to feed by the mouth, only very small quantities of predigested food should be given at a time. The best way to keep the strength up is to feed by the rectum, food being fairly readily absorbed from it; and the bowel should, in these cases, be regularly washed out, once a day at least, so as to prevent irritation, and a weak solution of cocaine may be used for the same purpose. The thirst of peritonitis may be relieved in a similar manner, but small pieces of ice may also be given by the mouth from time to time, or hot water be occasionally sipped, but the stomach is not to be filled with either one or the other, though an occasional long drink may be permitted for the definite purpose of washing out the stomach. This treatment makes the patient far more comfortable than keeping him from all drink by the mouth. No absolute rule can be laid down for every instance of any form of disease, and it is usually best to follow the inclinations or instincts of the patient where they are repeated and urgent, and where no obvious harm can be done. It is often subjecting the patient to the greatest torture to keep him entirely without liquids by the mouth, and the bodily and mental anguish so produced must be bad for anyone suffering from a depressing complaint like that which we are discussing. A spray of a weak solution of boracic acid to the mouth is often very grateful to the patient, while on the other hand it removes the fœtor which is commonly present in the breath of patients afflicted with peritonitis. If swallowed it will act as an admirable antiseptic to the stomach, &c. This may also be assisted by cleansing the mouth from time to time with some antiseptic solution, this at the same time helps to relieve the thirst. For this purpose 1-80 carbolic acid, or glycerine and water, are excellent.

### The Mechanism of Movement.

### VIII.—THE HAND.

The study of the general shape and formation of the bony structure of the body must also include the hands and feet, the extremities of each pair of limbs respectively.

The limbs in man and in all vertebrate animals are less important than the trunk or the head in the structure of the body on account of the organs contained in the latter. The head could not be removed or the trunk cut in pieces without life being destroyed, but not only one limb, but even all four may be lost and life not endangered necessarily. This proves that however important may be the functions performed by the limbs they are secondary to those of the trunk and head. The limbs are merely additions to the trunk to fulfil special purposes. The limbs are arranged in pairs in man and nearly all animals, but in animals lower down in the scale the limbs are placed not one beside the other, as in man, but one in front of the other.

Each limb is divided into three main parts—the "girdle," by means of which it is fastened to the trunk; the "shaft," between the girdle and the extremity, giving it length; and the extremity of the limb, the foot or hand respectively, which is the part which distinguishes or specializes it. The girdle and shaft are secondary in importance to the foot or hand, and their only use is to bring the hand or foot into the best position for carrying out the function of the limb.

The primary use of the upper limb or hand is to grasp or hold, what surgeons call "prehension," and a secondary use is as an organ of progression. We do not, as a rule, use our arms for this latter purpose, except to swim, or climb a tree, or scale a wall or rock. In this respect man differs from other four-limbed animals, in which the chief use of the fore limbs (corresponding to our arms) is not primarily to grasp or hold, but to support, and to carry from place to place. The modifications nature has made in the shape of the fore limbs in various animals depend on whether movement is to be rapid or not; this is well shown in the skeletons of the fore limb of the horse and elephant. In the first the formation promotes speed, whilst in the elephant the progress is very slow.

The fore limbs in animals are intended in the second place for prehension, or grasping and holding. Apes and squirrels can hold things with their fore limbs, whilst cats and lions use their fore limbs to strike their prey and also to hold it. Thus the primary and secondary uses of the fore limbs are exactly reversed in man and in animals.

As regards the lower limbs of man, their primary use is entirely different from that of the upper ones, viz., as means of support and progression. As a secondary use the lower limb can be trained to grasp or hold by means of the toes; but it rarely, if ever, comes naturally, and has to be acquired by long and painful practice. Thus the upper limb, or arm, is the general means by which we grasp or hold, and the hand the special means by which we accomplish it; and similarly with the leg. The foot is the part of the lower limb which enables us to move.

Though other animals have hands, it is only in the human hand that we find a perfect type, and no other animal accomplishes anything at all approaching the marvellous work accomplished by the hand of man. The human hand is composed of three principal parts, the wrist, which connects it with the arm; the palm, or lowest part of the hand proper; and the fingers, the most important part for the act of manipulation. Man has five fingers, and this is the highest number of fully developed fingers possessed by any mammal, though in some cases rudimentary fingers are found above this number. Each finger has a special name, the first and most important being the thumb, the strongest, thickest, and most active of all. The Romans recognized its special properties, and called it the pollex, meaning power, and another title they gave it was one meaning the "instructed," or skilful finger, and apparently they thought the skill of the hand depends on the thumb. We also show our appreciation of the power possessed by the thumb in such phrases as "under his thumb," to express the complete control of one person over another.

The Esquimeaux have such regard for the thumb that there is a prevalent notion amongst them that the woman arose from the thumb of the man, though whether this shows most respect for the thumb or contempt for the woman is difficult to say.

Though the thumb has the advantage in strength, thickness, and activity, it is wanting in dexterity, and hence a person who is clumsy is said to have "fingers that are all thumbs."

The index, or first finger, being the one with which we point or beckon, is named "index." It is the most movable of all, and is consequently used to beckon or to point with. The middle finger is the most important member of the hand because of its greater length.

The third, or "ring" finger has nothing special about it beyond being that on which custom prescribes that the wedding ring shall be worn. This custom has existed from early times and arose from an erroneous idea that a nerve runs direct from this finger to the heart.

The fourth or "little" finger is the shortest and least important of all.

It is curious that the fingers have never been seen of the same length, though the relative lengths of the first or "index" and third or "ring" finger vary, whilst the middle finger is invariably the longest and the little finger the shortest.

The hand possesses some very striking properties, such as great flexibility, elasticity, mobility, and sensitiveness. The elasticity, flexibility, and mobility of the hand are all largely due to the number of bones and consequently of joints that it has, as well as to the special arrangement of its muscles and tendons. The hand contains 27 bones, arranged in three groups, those of the wrist, palm, and fingers.

The bones of the wrist, eight in number, are called "carpal." The five in the palm, called metacarpal, are longer than the others. Fourteen bones form the five fingers, the thumb having two and each of the four fingers three bones, called collectively "phalanges," because they are arranged in rows like soldiers in the Greek phalanx. Thus the third division of the upper arm, viz., the hand, possesses alone far more bones than both the other parts put together. In the so-called girdle there are but two bones, the shoulder-blade and collar-bone; and in the shaft only three, the strong upper bone or humerus; and two in the part of the arm below the elbow, the radius and ulna.

The movement of the entire hand takes place at the wrist joint, and here the whole hand can even move forwards for a considerable distance, or be straightened out until it is in a line with the fore-arm, and then bent backwards; or the hand can be moved from side to side, to bring either the thumb or the little finger nearly up to the corresponding side of the fore-arm. All these can also be combined or continued into each other, and hence the varied range of movement which is so important to the usefulness of the hand.

### Marriage.

At the Bath Hotel, Glasgow, on the 5th inst., Nurse Weir, charge nurse of the Glasgow Training Home, was married to Dr. J. Anderson Robertson. The Very Rev. the Dean of Glasgow officiated. The bride's party was largely composed of sister nurses. The probationers appeared in uniform. The gifts to the bride included a handsome silver tea-service from the senior nurses, and a lovely set of china from the probationers at present in the home.

### Our American Letter.

The tendency to increase the length of training for nurses here is very marked, and now Danbury Hospital has decided to give a two years' course to its graduates.

Nineteen pupils recently gained their diplomas at the Training School for Nurses connected with the Philadelphia Hospital, several interesting addresses being given on the occasion by Dr. Weir Mitchell, Dr. James Tyson, and Mr. Gardner. The head of the Training School, Miss Marion E. Smith, was warmly congratulated on her successful management of the entertainment which followed the ceremony, and Mr. Lambert, president of the board, took a kindly interest in all the arrangements.

A two years' course is enforced at the Clifton Springs Sanitarium Training School for Nurses, and the first class of graduates received their diplomas this spring.

Miss Mary Leary has succeeded Miss A. L. Alston as Superintendent of the Mount Sinai Training School. Miss A. L. Alston has left a record of admirable work during her seven years in office, and takes good wishes from many friends to the Sanitarium which she proposes to establish in New York City very shortly.

The diplomas awarded by the Cleveland Training School for Nurses in April were received by twelve graduates, costumed in cream coloured Oxford gowns and "mortar boards." The twenty-five young ladies at the Training School, 163, East Thirty Sixth Street, New York City, were content on a similar occasion to wear a pretty hospital uniform of white aprons and caps and blue dresses.

Miss Elizabeth Sumner has been appointed Matron of Burbank Hospital, Fitchburgh, Mass. She was previously at St. Luke's Hospital, New York.

*The Trained Nurse* for July contains the announcement of Miss Isabel Hampton's departure from Johns Hopkins Hospital, to be married in England. The loss of such a superintendent cannot fail to be deeply regretted in the training school which she has organised so ably. The same journal contains some excellent articles on nursing matters, as well as "A Nurse's Day in a Hospital," by Miss Mary Agnes Snively, which is almost identical with "A Nurse's Life in Canada," printed in The Hospital of March 25th, 1893.

A new hospital is under contemplation in New York to be devoted entirely to the treatment of phthisical patients, and a hospital for coloured people only is discussed at St. Louis.

### Asylum Notes.—Ireland.

At a meeting held last week by the governors of the Richmond District Lunatic Asylum, two of the attendants were reported by the medical officer, Dr. Connolly Norman, for refusing to carry out his orders. "He did not wish to make these reports, but when a case of refusing to carry out his orders on a medical point occurred, he could not see how he could possibly overlook it," says the *Freeman's Journal*. And again: "Dr. Norman said he believed the nurses did their best to evade his orders." A discussion ensued, and Miss Breen was called before the Board, when she stated that in the thirteen years she had been an officer she had tried to do her best. "They" (Miss Raleigh and herself) "could not possibly carry out the doctor's orders without having to strangle the patient." Eventually, it is said, the nurses were reprimanded by the chairman, and warned against a repetition of the offence. The maintenance of discipline at the Asylum was thus enforced by the governors, but it did not transpire whether the female officers were qualified attendants and trained nurses, or if the latter word is merely a title of courtesy.

### Notes from Germany.

By A Visitor.

The heads of German public schools are busy selecting such pupils as stand most in need of change of air for a month's holiday in the country. Unfortunately all those who need it cannot be sent away; many a tired, fagged child must stay at home for want of the necessary funds, but for them two pints of new milk are provided daily by many of the charitable scholastic institutions.

Those who go into the country are placed under the charge of teachers who accompany them, and many pleasant excursions are planned by these self-sacrificing men and women for the benefit and enjoyment of the little ones who have perhaps never before enjoyed a romp in the woods, nor known the delight of plucking wild flowers, though poverty is not as extreme in Germany as in England. Many a little one fairly jumps for joy at the mere prospect of this coveted holiday, and the mother's face lights up with thankfulness when anxiety on account of her child's health is thus removed from her shoulders. Cheerfully she sits up at night to mend up the old frock and re-trim the shabby straw hat. She has her reward when she goes to the station to meet the child on her return, and sees the rosy, sunburnt face at the window looking out for "mother."

All children over six years of age must attend school at seven in the morning in summer and eight in winter, though on summer afternoons, should the weather be very hot, they are exempt from attendance.

### Our Examinations.

We have had a variety of answers to our question for June, and some of them are very good, two or three particularly so. Unluckily there is a great tendency amongst competitors to confound the nurse's duties with the doctor's responsibilities, and several otherwise excellent papers have been condemned because of their suggestions as to medical treatment, whilst insufficient description are given to precautions as to the handkerchiefs and the other homely, but most important, nursing details.

Question for June.

What precautions should be taken during and after an attack of measles, by the nurse in charge of the patient?

Prize Answer.

A nurse attending a patient suffering from measles, however mild the attack, should be sure to keep him warm, and especially out of draughts, to prevent any sudden chill. If his temperature be above 99 deg., he should be at once put to bed, all superfluous furniture should be removed from the room, which if possible should be large, and at the top of the house. A coal fire assists considerably in ventilation, and the window should be open night and day, at least about three inches from the top. The patient should be kept very quiet, with his eyes well protected from the light by drawing the blinds down in the daytime, and using a shaded lamp or candle at night. A chart should be kept recording his temperature, pulse, and respitation night and morning, also the nature and number of stools a day. The chief dangers in severe cases arise from pulmonary complications, therefore any cough and any rise of temperature or extra rapidity of pulse and respiration should be reported to the doctor.

The floor should be washed over daily with carbolic lotion, 1 in 60, and a sheet dipped in carbolic lotion, 1 in 40, should be hung outside the bed-room door (one inside also is advisable), to protect other members of the household. All soiled linen should be placed in carbolic lotion, 1 in 40, before being sent to the laundry. It is really better for some woman (without children) to come to the house to do the washing whilst measles is prevalent. When the patient is quite well, has had a disinfecting bath, and the doctor has pronounced him free from infection, the clothes and everything used during the illness should be left in the room, and thoroughly disinfected with sulphur fumes. After the patient is well enough to mix with other people great care should still be taken to avoid chills, as pulmonary mischief may even then set in. Nurse Eleanor Johnson.

### Imagination and Emotion.

By An Experienced Mental Nurse.

In speaking of the first insidious beginnings of mental disease and of the way in which we can best fortify ourselves against them, we saw that self-control may be of two kinds, outward and inward, or perhaps it would be more correct to say that it may be of two degrees, being partial or complete. It may be superficial self-control which checks unconsidered words and unseemly actions, and which is by no means easily acquired, or to be lightly esteemed; but our real source of strength is that deep rooted self-control which holds the thoughts and the emotions themselves, not merely the expression of them, in constant check.

There is no doubt that the outward composure which the rules of society require every well-bred person to cultivate, is a very good and useful quality, and it is not too much to say that most people consider it sufficient for all the ordinary purposes of life. But if they only knew it, this outward restraint costs them far more effort in the long run if it is not accompanied by the corresponding restraint of the passions. For these, if not continually held in check, naturally strive for outward expression, and the result is that the moral life is a perpetual inward conflict. True self-restraint gives rest to the mind, and a mind at rest is a mind strongly fortified against the attacks of disease.

On the other hand, where control of the emotions themselves is not practised, control of their outward expression merely is much more liable to break down in case of a sudden shock, or of a severe and prolonged strain upon the nerves. None of us dare say that his mental armour is proof against the possibility of disease, but certainly those who firmly hold the reins of their passions have much more chance than those who inconsiderately indulge them. I suppose that if all mankind could learn a real self-restraint, insanity might become a rare disease, the result of accident, instead of becoming year by year more terribly common.

One of the most troublesome and unruly gifts that a man can be endowed with is a vivid imagination. It is hard to say whether it is a source of more pleasure or pain, but this depends on the temperament of the individual, and on his capacity to guide and check his imaginative power. Unguided, it is a fruitful cause of suffering to its possessor, and to others beside himself. It is, in its nature, unpractical, unreal, and prone to exaggeration. A highly imaginative person, who has not counterbalancing practical qualities, is apt to look at things as they might be, instead of as they are, to magnify both his fortune and his misfortune, to be over-sanguine and over-despondent—in one word, to become unreal—to look at life from a distorted point of view. His imagination has got the better of his reasoning power.

A class of girls was once asked the question, "What is the use of the imagination?" And they found it not so easy to answer as perhaps they thought at first that it would be. It is true that in art, in literature, and in the sublime discoveries and inventions of genius, the imagination plays an important part; but there are many ordinary mortals who, while gifted with a lively imagination, have not the talents or the capacity to distinguish themselves by any remarkable achievement in art or science. To this numerous class of persons of what use is their imaginative power, which oftentimes seems more of a curse than a blessing?

It can certainly afford them the doubtful and dangerous pleasure of day dreaming; but it is against this very practice that I would utter a strenuous protest. There is nothing more weakening—I will go further and say more degrading—to the moral and intellectual nature than this habit of contemplating things that might be, but are not, of re-arranging the circumstances of our lives as we should like them to be, and suiting our mental attitude to these imaginary circumstances instead of to our real surroundings. The indulgence of day dreams distracts our thoughts, cripples our usefulness, and relaxes, so to speak, all the fibres of the mind; when the rude hand of calamity shatters the visionary fabric the nervous system is too often shattered with it.

What, then, is the legitimate use of the imagination for the average mortal? I would say, for the quickening of sympathy. It enables you to put yourself in another's place, and to enter into his feelings, even when his character and his circumstances are totally different from your own. And what wiser or nobler use could we find for our imaginative power than this—to enter more fully into the difficulties and temptations of those around us, to sorrow more unaffectedly for their sorrows, to rejoice more unselfishly with their joys?

Others, again, who are not troubled with an excess of imagination, have what is called an emotional nature, and what might, quite as correctly, be called an hysterical nature. I have no hesitation in saying that an emotional nature, as commonly understood, is a selfish nature. Unselfishness prompts us to the control of our emotions, which, as a rule, are not interesting to anyone but ourselves.

Uncontrolled emotion exhausts our nervous force; it is a traitor in the camp, which invites the approach of the foe, and throws open the doors of the citadel. In no other way, perhaps, can disease so surely take hold upon the brain, as by means of that mysterious and far-reaching complaint of hysteria. It is here that the line between sanity and insanity is most difficult to define, and one is tempted to say that a person of hysterical disposition is never really sane. From hysteria to hysterical mania is but a step, and sometimes a marvellously easy one.

But a person of hysterical tendencies may be aware of it, and anxious to control it. In this case the repression of outward signs of emotion is a great help to the interior repression of the emotion itself, while to give way to the outward expression of it will increase the actual violence of the emotion.

The reasoning powers may also be increased by cultivation. A habit of pausing to reason about any event or circumstances which causes us grief, annoyance, or terror, will in time enable us to regard with indifference the trifling and unavoidable worries of life, and to bear with fortitude its greater calamities. Reason gives us the power of seeing things in their due proportions, while emotion always tends to exaggerate. It is just that incapacity to reason, or even to listen to the reasoning of others, that makes a hysterical person, for the time being, practically insane.

When we feel this tendency in ourselves, or know that we are likely to have inherited it, we may counteract it to a great extent by this exercise of reason, and by constant occupation of the mind. Merely mechanical occupations which can be carried on while the mind is roaming at large, are useless as checks either to day dreaming or to waste of emotion. Work which will occupy the thoughts and exercise the intellect—above all, work which will take us out of ourselves, and give us our chief interest in things which are not our own—is the only real antidote against a morbid condition of mind.

Sometimes the imagination plays an important part in the vagaries of hysterical people, as when the cause of the emotion, or even the emotion itself, is partly imaginary. If they are entirely imaginary, we may rightly call the person insane; and in either case the unreal nature of the emotion may be easily proved by stimulating the imagination with some fresh idea, when the previous current of thought will be quite forgotten.

In this age of overworked brains and highly strung nerves, how shall we escape these impalpable yet serious dangers which threaten our mental equilibrium? Must we succumb to the fatal influences of heredity, of education, and of circumstance, or is there a remedy within the reach of us all?

I think there are, at least, preventive measures, and I have endeavoured to point out what they are. To exercise, in all matters, great and small, that faculty of reason that none of us were born without; to cultivate the imagination chiefly as an everflowing spring of comprehending sympathy; and not to waste our emotions on the unimportant occasions of life, or on occasions which have never yet arisen, and probably never will arise.

It is a true saying that "Self control is wisdom's root." It is also the basis and the safeguard of mental as well as bodily health.

### White Phosphorus and Necrosis.

Mr. Percy Wyndham has drawn up an interesting memorandum embodying the laws and regulations in force in Germany and Prussia with regard to the manufacture of lucifer matches. It seems that in 1879 the Reichstag petitioned the Imperial Chancellor to prohibit the use of white phosphorus in the manufacture of matches, and at the same time to impose an increased import duty on matches. A commission of specialists was accordingly appointed to inquire whether in order to check necrosis such a prohibition was necessary, or whether the desired result could not be attained by a decree regulating the construction and management of match factories; prohibition of the use of white phosphorus was obviously the simplest means of stamping out the disease, but as so stringent a measure would unduly interfere with trade and expose the export of German matches to unfair competition with that of other countries where no such prohibition obtained, and as, finally, many factories could be instanced where no cases of necrosis had occurred owing to proper precautions having been taken, it was determined to regulate the use of white phosphorus, and the efforts of the commission resulted in the law of May 13th, 1884.

The chief provisions of this law were: (1) That the manufacture of lucifer matches with white phosphorus shall only be conducted in buildings which are entirely devoted to the manufacture of matches; and (2) that juvenile workmen are not to remain in rooms in which (*a*) the phosphorus is being mixed, (*b*) the wood is being dipped, or (*c*) the wood which has been dipped is being dried, and (*d*) children are not to remain in the rooms where the matches are first placed in boxes and packed.

This law was followed by a declaration of July 11th, 1884, which decreed that each of the four operations specified above must be conducted in special rooms, only immediately connected with each other and not with other workrooms or dwelling-rooms; direct communication is, however, permitted between the room where the wood is dipped and where it is laid by, and also between the room where the matches are first placed in boxes and packed and where the finished goods are stored. Minute instructions follow as to the structural arrangements and ventilation of the rooms where these operations take place, the daily cleansing of such rooms, the wearing by the labourers employed of special aprons or overcoats, and the provision of separate cupboards for hanging up the ordinary clothes and the working clothes. Workmen are not allowed to bring food into the workrooms, nor to eat there; before taking their meals, in quite distinct quarters, they must wash their hands and rinse their mouths, and finally no persons are admitted to work in these factories unless they have received a certificate from a certified doctor that they are not suffering from necrosis, and that they are not particularly liable to this disease. Monthly inspections of the workmen by a certified doctor were also ordained, and the employer was compelled to keep a register showing the length of service of each of his men, together with the observations of the factory doctor and their date.

On July 8th, 1893, these regulations were re-enacted with but slight alterations, the most important being the substitution of quarterly for monthly examinations of the workmen, and from this last regulation it appears that the observance of special rules is sufficient for the suppression of necrosis without the prohibition of the use of white phosphorus.

### The National Home Reading Association.

A Visit to Salisbury.

Glorious weather and a cordial welcome from "town and close" combined to make the summer assembly of the National Home Reading Union, held this year at Salisbury, a signal success. The meeting was opened by the Mayor of Salisbury, who held a reception for the members in the Council Chambers on Saturday evening, June 30th, and the proceedings terminated on July 8th by a special sermon in the Cathedral, preached by the Rev. the Provost of Eton.

From the 2nd to the 6th inst. several lectures were delivered daily in the County Hall, the main object of them all being to complete the course of study the members have pursued during the past session, by presenting vivid pictures of life in Saxon, Scandinavian, Norman, and Augevin England, with special reference to the architectural evolution of castles, towns, and churches, to the university, cloister, and Court life of mediæval scholars, to the growth of musical taste in Celt and Saxon, and to the effects of the conflicting characteristics of the various races whose fusion formed the English nation.

The neighbourhood of Salisbury abounds in historical associations, and the afternoon excursions, admirably organised by the local committee, were planned to afford practical illustrations of the morning lectures. Stonehenge, Old Sarum, the picturesque village of Amesbury, where once stood Queen Elfrida's nunnery, were visited on Monday, the 2nd; Downton "Moot"—Longford Castle, the following day. The Very Rev. the Dean of Salisbury and Mrs. Boyle entertained the members in the beautiful garden of the Deanery on Wednesday afternoon. A visit to Wilton House and a conversazione in the Salisbury Museum occupied Thursday, while Friday afternoon was devoted to the inspection of one of the grandest Norman abbeys remaining in England, that of Romsay, associated with the name of Edgar Atheling's sister, Matilda, who, after the Norman conquest, lived here in seclusion until her marriage with the conqueror's son, Henry I.

The whole of Saturday was spent in an expedition to General Pitt Rivers's fine ethnological and archæological collections at Rushmore and Farnham.

In striking contrast to the subjects of lectures by Mr. York Powell, Mr. Tanner, and Mr. Baldwin Brown, all university professors of Norse, Saxon, or Early English lore, were the scholarly disertations upon the poetry of Browning, by the Dean of Salisbury; an eloquent account of Samuel Johnson, by Professor Jebb, M.P., given on the evenings of the 2nd and 6th inst. respectively. Apparently all Sarum mustered to hear Sir Robert Ball "On Some Recent Discoveries about the Sun," on Wednesday evening the 4th, which lecture, together with four admirable ones on botany delivered during the course of the week by Mr. Seward, of Cambridge, and demonstrations on Stone-Age remains in the museums, by Professor Hughes, formed the scientific instruction provided for the assembly.

We noticed several nurses in uniform among the audiences at the lectures, and we mentally congratulated the wearers of the trim bonnets on their wisdom in taking advantage of opportunities for self-culture beyond the professional range of studies to which some are inclined to confine their energies.

A change of occupation is often the truest form of rest, and nurses have to guard against a tendency to become narrow.

Courses of reading can be followed in any language or upon almost any subject, and all particulars will be gladly supplied by the Secretary, Surrey House, Victoria Embankment, W.C.

### Women's Work.

### HEALTH LECTURING IN RURAL DISTRICTS.

By a Lecturer.

As I have now been lecturing on sick nursing and hygiene for over a year in various counties in England, it is possible that some of my experiences may be of use to those about to take up similar work.

I am a fully-trained nurse, and I certainly consider that no woman without such training is competent to lecture on sick nursing, however well she may be able to speak. Cottagers, though perhaps themselves ignorant, form decidedly critical audiences, and unless a lecturer shows that she herself has accomplished the things she is telling them how to do, she will hardly get an attentive hearing. Not only hospital but district training is needful, that is to say, experience in nursing the poor in their own homes. A few illustrations from life will impress rural audience, in a way that no amount of book learning could possibly do. They like to say, "Ah! that's true now, she knows what she's a-talking of."

But with all her training a competent lecturer must expect to be considerably nonplussed sometimes. For instance, after quoting impressively from Miss Nightingale the first canon of nursing—the first and last thing upon which a nurse's attention should be fixed is this: "To keep the air the patient breathes as pure as the external air without chilling him," and enlarging on the benefit of open windows and clear fire-places, a voice audibly says, "All very fine, but our windows don't open, and we ain't got no fireplaces!" Also suppose the subject is cleanliness of the skin. A lecturer remarks: "Now you really cannot expect to have perfect health unless the skin is washed all over every day. Of course I know to many of you a bath is out of the question, but the skin can be kept quite clean with a small quantity of warm water and soap and a piece of flannel. Water costs nothing." "Don't it though!" interrupts somebody, "we ain't got no water except a pond of dirty stuff swarming with insects. We have to boil and strain it before we can even use it for scrubbing. In summer we have to fetch the water the best part of a mile!" It really seems at last almost a mockery to lecture on the principles of health when even air and water are beyond reach; but, of course, every audience is not so badly circumstanced.

More often than not when I speak of feeding infants, and I say, "No food but milk should be given," some lady asks, "Is there really no substitute? The people here cannot get milk; all the farmers send theirs to London."

Poultices generally excite more enthusiasm than anything else. A poultice that will roll without sticking, and allow of being thrown from hand to hand without damage, is looked upon as of the nature of a conjuring trick. Frequently a second poultice has to be made to show that there is no deception or "trick" in the manufacture, and then these are taken home to be shown about and looked at over and over again.

Frequently the lecturer is accused of being personal; some old woman goes out in a huff, and it is discovered afterwards that she fancies "some 'un has been talking, or how could the nurse know that she kept her boxes of onions under her bed?" Besides these rustic criticisers, the lecturer is sometimes confronted with a dozen or more of young ladies, who come smilingly forward and say, "We adore nursing; we are so much interested. You know we have all got St. John's Ambulance certificates!" Then the lecturer may rest assured that they all feel confident of knowing more than she does herself, and that they will all sit and smile patronisingly on her from the front row. A. E. I.

### Wants and Workers.

Sister Miriam and Nurse Charlotte are cordially thanked by Sister Winifred for their kindness in copying the verses for her.

### Queen Victoria's Jubilee Institute for Nurses.

Her Majesty has been pleased to approve of the following names being added to the roll of Queen's nurses for nursing the sick poor in their own homes:—

Superintendents.

Dorothea M. Oldham, Rural District Branch; Mary T. Robson, Bermondsey; Jane Brown, Leeds; Laura A. Wing, Coventry; Alice F. Wathen, Northampton; Annie B. Harris, Holbeck; Emily Smythe, Londonderry.

Nurses.

England. — Millicent Goodwin, Haggerston; Mary Bladsworth, Hammersmith; Merimna Lloyd, Helen Murphy, and Elise de Neuman, East London; Alice Watson, Rotherhithe; Helen M. Gibbs, Addlestone; Mildred Hodges and Mabel A. Spackman, Aldershot; Pattie Metzger and Georgina V. Young, Bramley; Violet E. Wood, Bishop Auckland; Amy P. Shaw, Theresa J. Lesser, and Elizabeth J. Oliver, Bolton; Rose Lesser and Elizabeth T. Folk, Coventry; Mary H. Morton, Darwen; Jane Barnes, Darlington; Arabella M. Wyatt, Droylsden; Mary E. Walker Mary Parry, Fanny E. Jacob, Mary Proudfoot, Maude Proudfoot, Alice Jackson, and Mary Sewell, Leeds; Ada Arrowsmith, Florence W. Lett, and Alice Ada Seed, Liverpool; Emily Baxter, Rose E. Daly, and Zella Johnson, Manchester; Kate Isherwood, Portsmouth; Annie Marie Hicks and Zella T. Williams, Southampton; Alice T. Warwick, Torquay; Harriet E. Bailey and Mabel Prosser, Tunbridge Wells; Edith E. Moore, Warwick; Alice Pickering, Wellingborough.

Wales.—Elizabeth T. Platt and Sara Podmore, Cardiff; Alice A. Bowles, Llandovey; Eleanor M. Franks, Merthyr Vale; Annie Michie, Pembroke Dock.

Rural District Branch.—Mary B. Duncan, Chorlton; Blanche M. Glover, Sutton; Martha T. Loane, Buxton; Sarah Snell, Langport; Frances M. Stiffe, Bloomsbury; Elizabeth Walden, Wribbenhall; Betsey H. Wherrit, Barkstone; Florence M. Wilson, Freshford; Lucy A. Yeatman, Hoo.

Irish Branch.—Josephine Ellison, Honoria M. Law, and Elizabeth C. Ryder, Dublin; Sara Dunne and Lina Kruysee, Londonderry; Kathleen Plumb, Omagh; Annie E. Glynn, The Rosses.

Scottish Branch.—Barbara Bizzart, Isabella M. Davidson, Hughina B. Fraser, and Elizabeth E. Thomson, Edinburgh; Catharine A. O'Sullivan, Glasgow; Annie E. Steen, Port Glasgow; Annie L. Day, Aberdeen; Emily D. Walton, Beith; Cecilia B. Fawley, Johnstone; Rebecca Camp, Monaive; Isabella Bayne, Paisley; Mary F. Wade, Peebles; Beatrice Græme and Katharine S. Macqueen, Perth; Alice J. M. Rumsey, Selkirk.

### A Case for Assistance.

In connection with the appeal made in The Hospital of June 30th on behalf of a nurse, Miss Bell and Miss Farrow write from Liverpool Homes for Aged Mariners, Egremont, Cheshire, "to acknowledge with gratitude the further sums of 5s. from Miss W.; 2s. 6d. from L. S.; 2s. 6d., M. G.; 5s., H. L. W.; making a total of £2 up to present date."

### Notes and Queries.

### Queries.

(97) *Insane.*—Is there any asylum for the insane in Norfolk?—*Enquirer.*
(98) *Book.*—Please give me the title of the book on surgery suitable for nurses.—*H. M.*
(99) *Training.*—Information required.—*B. M. B.*

### Answers.

(97) *Insane (Enquirer).*—Heigham Hall, near Norwich.
(98) *Book (H. M.)*—Is "Surgical Ward Work," by Alexander Mills, what you require? It is published by the Scientific Press, 428, Strand.
(99) *Training (B. M. B.)*—Get "How to Become a Nurse," by Honnor Morten, and write to Matron at Guy's Hospital, the London Hospital, or the Royal Free Hospital, Gray's Inn Road, on the subject you mention.

☞ For Everybody's Opinion, Reading to the Sick, Book World for Women and Nurses, &c., see page clix. et seq.

### Our American Sisters.

A WEDDING AT WESTMINSTER.

A STORMY night and cloudy morning were succeeded by a gloriously bright noon on July 11th, the wedding day of Miss Isabel A. Hampton.

Miss Hampton's brilliant career is well-known to nurses, and the organisation of the training school for nurses at Johns Hopkins Hospital, Baltimore, remains a permanent memorial of her administrative abilities. She received her training at Cook's County Hospital, Chicago, and during fifteen years she has laboured for the advancement and perfecting of the profession of trained nursing in America.

Miss Hampton, herself the busy head of a model school, has yet spared time to write a book for the aid and guidance of superintendents and nurses, which is quite a unique work, dealing exhaustiveiy with every branch of skilled nursing. Setting before her sisters a high ideal, she shows a lofty standard, which must also be attained and not lowered by those following in the footsteps of so able a teacher and guide.

Hearing of her long years of noble work, it was almost a surprise on Wednesday to see the fresh fairness and youth of the gracious and stately bride who entered the quiet old church at Westminster on the arm of Mr. Merritt, who with his wife had come purposely from Scotland to be present at their friend's marriage.

Dr. Hunter Robb, of Cleveland, Ohio, was accompanied by his two brothers, and English as well as American friends awaited the bride and bridegroom, with Miss Rogers and Miss McLean, of Baltimore, who came to England with Miss Hampton three weeks ago; Mrs Bonham Carter (a fellow-countrywoman), Miss Amy Hughes, Mr., Mrs., and Miss Burdett, Lady Colin Campbell, and others.

The ceremony was performed by Archdeacon Farrar, D.D., rector of the parish. Mrs. Merritt gave away the bride, whose dress, of a bluish grey tint, was very becoming. It was trimmed with russet velvet, and the bonnet corresponded with it, but the delicate white bouquet, with long white ribbon bows, gained more attention than any other detail, for everyone in the church knew that Miss Florence Nightingale had just sent it. A long interview took place on Sunday between the latter and Miss Hampton, and the meeting cannot have failed to interest both.

After the wedding ceremony was over most of those present repaired to the vestry to convey their congratulations to the bride and bridegroom, who were starting to Oxford. Clevedon has been lent by Mr. Astor to Dr. and Mrs. Robb for their honeymoon, and they will inhabit the bridal suite of apartments which the Duke of Westminster placed at the disposal of the Princess Christian on the occasion of her Royal Highness's marriage.

Clevedon is too famous a palace to need any eulogies, and is, indeed, too beautiful in itself and its surroundings to be easily described.

Miss Hampton's time in England has been too short and too fully engaged to allow of her seeing more than a small proportion of those who would gladly have made acquaintance with such a leader of nursing. However, it has caused much pleasure to many of those who admire and love her for her work that she should have chosen to be married in the heart of London, and if our American sisters regret the arrangement we trust they will not grudge us this small part of Miss Hampton's time, as she and her husband are returning in the autumn to make their home where Dr. Robb has been appointed Professor of Gynecology, at the West Central University, Cleveland, Ohio.

The marriage certificate runs as follows: "Parish Church, St. Margaret's, Westminster, July 11th, 18[illegible]4. Hunter Robb, batchelor, Doctor of Medicine, London, son of Thomas Robb, deceased, married to Isabel Adams Hampton, spinster, daughter of Samuel Hampton. Witnesses: Walter Robb, Elizabeth R. Merritt, Henry C. Burdett, Amy Hughes."

### Appointments.

SOUTH LONDON DISTRICT NURSING ASSOCIATION.—Miss Susan Mary Warner has been appointed Superintendent of this Association. She was trained at Edinburgh Hospital and the Metropolitan and National Nursing Association, and was Superintendent of the Portsmouth District Nursing Association. Such excellent experience specially qualifies Miss Warner to hold the responsible post to which she has been elected, and we congratulate her on her appointment.

ST. MARK'S HOSPITAL, CITY ROAD, LONDON.—Miss Edith Helen Owen has been appointed Matron of this hospital. She was trained at the London Hospital, where she afterwards held the post of Ward Sister. We congratulate Miss Owen on her appointment, and wish her every success.

ROYAL BATH HOSPITAL AND RAWSON CONVALESCENT HOME, HARROGATE.—Miss Edith Sutcliffe, who has received the appointment of Matron at this Home, was trained at St. Mary's Hospital, Paddington. She was afterwards at the Bristol Infirmary, the Hospital for Women, Soho Square, and Matron of the Hospital for Women and Children, Leeds. We wish her continued success in her new work.

BOOTLE BOROUGH HOSPITAL.—Mrs. Annie Roberts, who was trained at this hospital, has recently been made Matron of it. She held the post of Charge Nurse in the same institution, and was Matron of Linacre Fever Hospital. Mrs. Roberts is therefore thoroughly conversant with the duties of the post which she has been elected to fill, and is already known and valued in Bootle.

WEST QUAY INFECTIOUS DISEASES HOSPITAL, SOUTHAMPTON.—Miss J. Ferguson has been appointed Matron of this hospital. She was trained at the Royal Infirmary, Glasgow, and holds a three years' certificate. She was nurse at the South-Eastern Fever Hospital, Night Superintendent of St. Olave's Union Infirmary, and Assistant Matron at the Worcester General Infirmary, and takes many good wishes with her to Southampton.

BERKELEY HOSPITAL.—Miss Hannah Darnborough has been appointed matron of this hospital. She was trained at the London Fever Hospital and Oldham General Infirmary, was Night Superintendent of Nursing at the Staffordshire General Infirmary, also Night Superintendent of the Scarlet Fever Department at Birmingham City Hospital. We congratulate her on her appointment.

BLACKBURN FEVER HOSPITAL.—Miss Elliott has been made Matron of this hospital. She was trained at St. Thomas's Hospital, and was Charge Nurse and afterwards Night Superintendent at Monsale Fever Hospital, Manchester.

### Minor Appointments.

SOUTH SHIELDS UNION.—Miss Kate Denton has been made Superintendent Nurse at the workhouse of this union, and her previous extensive experience well fits her for her new responsibilities. Miss Denton was trained at the Salop Infirmary, Shrewsbury, afterwards worked at the Hereford General Infirmary, was private nursing in Nottingham and Cornwall, Night Superintendent at East Lancashire and Blackburn Infirmary, also Charge Nurse at Halifax Union.

MOTHERWELL INFECTIOUS HOSPITAL.—Mrs. Mary Christie has been made Nurse-Matron of this hospital. She was trained at Paisley Infirmary, where she was sent by the late Mrs. Higginbotham, of the Sick Poor and Private Nursing Association, Glasgow, and was afterwards for five years and ten months nursing in connection with the Home, and for one year private nursing on her own account. We wish Mrs. Christie success in her new work.

### Everybody's Opinion.

[Correspondence on all subjects is invited, but we cannot in any way be responsible for the opinions expressed by our correspondents. No communications can be entertained if the name and address of the correspondent is not given, or unless one side of the paper only be written on.]

### DISTRICT NURSING.

"H. A. D." writes: Several letters have appeared in The Hospital upon the subject of district nursing. May I give an opinion? I do not pretend to be a judge, but this I do know, that were I sick and poor and in need of care, I should like to feel that my nurse had received thorough training, and also, if possible, I should like her character to resemble that portrayed in the following exquisite little verse of Halliburton's:—

Sweet the soul that finds her treasure
In the sight of others' pleasure,
Whose dear heart is always bent
On some gentle, kind intent;
Who, unwearied night and day,
Follows in the dear Lord's way,
Undefiled by evil's breath,
Through sweet life to sweeter death.

The time is not far off for us all when we shall know the need of loving ministrations. Till then, let us give to our poorer brethren of our best. Remembering our Lord's words, "Inasmuch as ye did it unto one of the least of these, ye did it unto Me."

### NURSE OR NEEDLEWOMAN?

"Nurse E." writes: I have recently heard many nurses like myself engaged in medical and mental nursing complain of the sewing they have to do when engaged in private nursing. Before entering on duty we are duly informed, either verbally or by letter, that "needlework must be done." Now no sensible nurse will refuse to mend the clothing required by her patient. Yet she is not called upon to mend for all in the house, or to do sewing for charitable purposes. If she has leisure she certainly wants fresh air, and, like doctors, needs time for reading, or she will be left behind in her profession. A nurse is expected to keep her patient from harm if it be a mental case; if a medical case, the care and responsibility are equal. The nurse is supposed to bring to the front all the experience she has obtained by a life of hard, earnest, and serious work, and to her the friends look for a possible cure to the patient left in her charge. This sewing is now so largely asked for that nurses would do well to consider the subject. And yet another complaint. In answering advertisements we send stamp "for reply," but no letter comes to say you have or have not got the place. Where does the stamp go? Perhaps some fellow nurses will say something on this subject.

*** Our correspondent is somewhat unreasonable in making a grievance of the work which she states is plainly set forth as part of the duty she enters upon. If the needlework had come as a surprise or as an "extra" which she had not undertaken to do, the case would naturally be different. We know that many trained nurses, when with a long case which does not need much attention, are glad to help the mistress of the household by voluntarily relieving her of odds and ends of work. Such kindly offices as these are often taken as a matter of course by a mere guest in a house where sickness or limited means, or both, make the burden of life lie heavily on the "house-mother. An attendant (by no means always "a nurse" in the modern meaning of the word) engaged permanently with a chronic case may well be expected to do a reasonable amount of needlework, and yet have time left for the reading which "Nurse E." considers she, "like the doctors," requires. We have always considered books to be a necessity in every life, and certainly should not limit their use to doctors and nurses. Whilst considering that incessant needlework would be an unjustifiable burden to be laid upon any nurse, we are yet confident that the best women in every profession are those least given to forget the old yet ever new "golden rule."—Ed. *T. H.*

### For Reading to the Sick.

### THE FATHER'S LOVE.

**Motto.**

The story without an end which angels throng to hear.
—*M. Tupper.*

**Verses.**

My God Thou art all Love!
Not one poor minute 'scapes Thy breast
But brings a favour from above,
And in this love I rest.
—*George Herbert.*

My God how beautiful Thou art,
Thy Majesty how bright;
How beautiful Thy mercy seat,
In depths of burning light.
No earthly father loves like Thee,
No mother e'er so mild,
Bears and forbears as Thou hast done
With me, Thy sinful child.
Yet I may love Thee too, O Lord,
Almighty as Thou art,
For Thou hast stooped to ask of me
The love of my poor heart. —*Faber.*

**Reading.**

Just as it is possible to be tired in body, so it is possible, and also common enough, to be tired in mind. Have you not felt this at some time or other? It may be that you are feeling it now as you lie on a sick-bed, and have so much time every day for thinking. Or perhaps you are able to sit up part of the day, and though you are cheered at the thought of being better, still your mind feels so tired all the same. Wearisome thoughts will rush in and out, not the thoughts you would like to have. You cannot think properly, nor pray properly, and you cannot find much comfort in the holy Bible words. All seems to go wrong.

And you are disappointed and out of heart because you have read in books about people who in sickness had such strong faith and sure hope, such sweet comfort in prayer and trust in their Saviour, you are disheartened and cast down, and then a sort of fretfulness comes over you, something like a child's fretfulness (for we are all like children when we are sick and tired) and you say "I will give it up, I cannot find rest for my poor weary soul. I suppose I am not religious after all, or else God has forsaken and forgotten me."

Are not these something like your thoughts? . . . The body affects the mind very much indeed, more than we who are in good health are perhaps aware of, and you must make due allowance for this. So do not be cast down, nor too ready to think you are not religious, simply because you cannot feel strongly.

Let us go quietly and sensibly into the matter, and take only one step at the time. First—

Do not expect a great deal of yourself. Do not weary yourself with long Bible readings, nor even long prayers; just take a very few words home. You would not give a tired child a long lesson to learn. Now your soul is tired, and God knows it. He does not want much from you, but sends you a few words of cheer to day.

These are the words: "The Father Himself loveth you."

Who is it that loves? It is your Father. Rest a moment on that thought. You are reminded of the time when you were a child, when your earthly father worked for you, and strove hard, and bore many things for his children's sake. You can recall all these, which were so many proofs of his love for you. Well your Heavenly Father's love at this moment is a little like that father's love, only far tenderer, far stronger, far more pitiful. No child can feel lonely nestling in his father's arms, and you are resting in God's arms now. "The Eternal God is thy refuge, and underneath are the Everlasting Arms." How beautiful is that promise! You are not alone nor forgotten; no, not for a single moment. —*C. M. Hallett.*

### The Book World for Women and Nurses.

[We invite Correspondence, Criticism, Enquiries, and Notes on Books likely to interest Women and Nurses. Address, Editor, The Hospital (Nurses' Book World), 428, Strand, W.C.]

The Great Eastern Railway Company's Tourist Guide to the Continent. Edited by Percy Lindley. (30, Fleet Street. 1894. Price 6d.)

This is the fifteenth annual issue of this illustrative and descriptive guide to Holland, North and South Germany, and Switzerland, and is published by authority of the Great Eastern Railway Company, which has done so much of late years to develop the route to the Hook of Holland, viâ Harwich. It is less a guide-book in the ordinary sense of the word, than an interesting description of the various places embraced within its purview, and is full of information. The illustrations are copious and good. The editor has wisely added a useful itinerary for cyclists, which cannot fail to be serviceable. The book can be easily put into the pocket.

The Continent, by the Queenborough Route, via Flushing. A Comprehensive and Unconventional Guide to the Continent. By H. Tiedeman. (London: Iliffe and Sons. 1894.)

We have looked through this handbook for "English and American Tourists Abroad," and can speak of it in terms of the highest praise. It is both comprehensive and unconventional, lucidly and methodically written, full of interesting and useful information given in the shortest compass. It really makes one long to be able to make use of it. Plainly and clearly printed, with excellent maps, charming photographs, and other illustrations, with the various routes more than intelligently laid down, it strikes us as being quite the best book of its kind that has ever come under our notice. The advice given in the preface and elsewhere is practical and well worth digesting, and intending travellers will find it of the greatest service. The price is 3s., a marvel, we think, of cheapness.

Young Sam and Sabina. By Tom Cobbleigh. 1 Vol. (London: Fisher Unwin. 1894.)

This is a most delightful addition to the Pseudonym Library, compressing as it does a remarkably large amount of story telling into a remarkably small space. The story is most original in its situation, and tells of idyllic village life at Middleney in Somersetshire. Middleney is described as a sort of inland island situated on a high rise of ground in the Somersetshire moors, and annually surrounded and isolated by the winter floods. The adult population in this damp arcadia consists of seven persons, two of whom are Young Sam and Sabina, the lovers of the story. Perfect peace reigns in the little society, for persons so dependent on one another could not afford to quarrel, and gossip is limited to neighbourly praise. Then comes discord one year with the snipe shooting and the appearance of Ashford, a young man from the nearest town, who amuses himself with flirting with Sabina. Being a lawyer's son, however, Ashford considers himself too far above Sabina in social position to make her serious proposals, and the matter goes no further than one-sided love making. The quiet little river where Ashford and Sabina meet in the evening during the following summer, to the scandal of Middleney, is very prettily described. Young Ashford comes and goes, and the breach widens between Sam and Sabina, until the next winter, when Sam gets caught and surrounded by the flood, and is rescued in a boat by Sabina, who had meanwhile overheard Ashford denying any serious intentions towards her. Sam's danger reveals to Sabina that it is he only that she really loves, and the end seems satisfactory to both of them. Side by side with the love story runs one of equal interest, in which Sam's father, old Christopher Grinter, and the widow Sophia figure. The only reasonable fault the reader can find in the little book is that old Christopher's character, so delightful in all other respects, is never quite cleared up in regard to some money transactions. Sophia has a convict son, to whom she sends money, with the aid of Christopher, who knows all the time that the son is dead, having escaped, been recaptured, and hanged. The reader is left in doubt as to whether old Christopher gave back the money or made a good thing out of it. "Young Sam and Sabina" may be considered as one of the greatest successes of this series, and possesses especial attractions for those acquainted with Somersetshire.

### MAGAZINES OF THE MONTH.

The National Review prefaces its contents with an excellent editorial commentory on the episodes of the month. The July number of this high-standard magazine contains much interesting matter. A chapter on Gogol, by Arthur Titley, shows a consummate study of the Father of Russian Realism on the part of his present biographer. This article is not only instructive, but literary; and though much else in "The National Review" claims our attention, this article especially attracts us. A member of the Bechuanaland Police gives a dispartial account of "Campaigning in Matabeleland," and succeeds in throwing some new light on a subject of which a plethora of accounts have already appeared.

The Humanitarian for this month displays the names of Sir Henry Roscoe, the Dean of Ely, Lady Burton, Lady Violet Greville, and "Ioto" as contributors. It is the best number of the magazine which has yet appeared. The fact that the authoress of "A Yellow Aster" contributes a story will especially recommend the present issue in some quarters, but other articles, upon perusal, will be found to lay greater claims to our interest.

"A Horrid Suspicion" in this month's Idler is an entertaining account of an incident happening to some people who kept "open house," as they described it, in the country, an incident which, whilst it might possibly occur under any circumstances, is fortunately not entirely in the realms of the probable. The incident is entertaining and well described, and the illustrations good. "Johnnyboy," by Bret Harte, is a capital little story commencing the present number of the Idler, which continues to maintain its character of a highly-popular magazine.

The Holiday Number of London Society is capital railway reading. The print is clear and good, and it is a handy volume as a "compagnion de voyage" Here are several short stories, complete in themselves; no serials, "to be continued" in our next, and worry our minds until they are continued. But, instead, we have five tales of fairly good length, by writers already well known to the public. We commend this volume as an excellent purchase for the holiday season.

### Books Received.

Drake, Driver, and Leaver.

"Asylum Construction." By George Bibby, F.R.I.B.

H. K. Lewis.

"Post Nasal Growths." By Charles A. Parker.

"Inebriety or Narcomania." By Norman Kerr, M.D., F.L.S.

**Periodicals and Pamphlets.**—The Girl's Own Magazine, The Boy's Own Magazine and Summer Numbers, The Leisure Hour, The Sunday at Home, The Therapist, Rural Hygiene (by Florence Nightingale), Reich's Medicinal Auzeiger, St. Nicholas, The English Illustrated Magazine, South Africa, The Cornhill Magazine, Cassell's Family Magazine, Guy's Hospital Gazette, Practical Points on the Hygiene of Ships (by W. Collingridge, M.D.), Food and Sanitation, The Tourist Guide to the Continent, The Beginner's Guide to Photography. The Humanitarian, Public Health, Food and Sanitation, Provincial Medical Journal, Vegetarian, In His Name, Leeds Hospital Magazine, The Zenana, Amateur Gardening, Annual Report to County Council of Stirling and Dumbarton.

